# A Systematic Review of MRI Studies and the “Emotional paiN and social Disconnect (END)” Brain Model of Suicidal Behavior in Youth

**DOI:** 10.1155/2023/7254574

**Published:** 2023-09-23

**Authors:** Olga Tymofiyeva, Katherine W. Reeves, Chace Shaw, Eric Lopez, Sepehr Aziz, Jeffrey E. Max, Tony T. Yang

**Affiliations:** ^1^Department of Radiology and Biomedical Imaging, University of California, San Francisco, CA, USA; ^2^Philip R. Lee Institute for Health Policy Studies, University of California, San Francisco, CA, USA; ^3^Department of Psychiatry and Behavioral Sciences, The Langley Porter Psychiatric Institute, Division of Child and Adolescent Psychiatry, Weill Institute for Neurosciences, University of California, San Francisco, CA, USA; ^4^Department of Psychiatry, University of California San Diego, San Diego, CA, USA; ^5^Rady Children's Hospital, San Diego, CA, USA

## Abstract

**Introduction:**

Risk of suicidal ideation and suicidal behaviors greatly increases during adolescence, and rates have risen dramatically over the past two decades. However, few risk factors or biomarkers predictive of suicidal ideation or attempted suicide have been identified in adolescents. Neuroimaging correlates hold potential for early identification of adolescents at increased risk of suicidality and risk stratification for those at high risk of suicide attempt.

**Methods:**

In this systematic review, we evaluated neural regions and networks associated with suicidal ideation and suicide attempt in adolescents derived from magnetic resonance imaging (MRI) studies. A total of 28 articles were included in this review.

**Results:**

After descriptively synthesizing the literature, we propose the Emotional paiN and social Disconnect (END) model of adolescent suicidality and present two key neural circuits: (1) the emotional/mental pain circuit and (2) the social disconnect/distortion circuit. In the END model, the emotional pain circuit—consisting of the cerebellum, amygdala, and hippocampus—shows similar aberrations in adolescents with suicidal ideation as in those with a history of a suicide attempt (but to a smaller degree). The social disconnect circuit is unique to adolescent suicide attempters and includes the lateral orbitofrontal cortex (OFC), the temporal gyri, and the connections between them.

**Conclusion:**

Our proposed END brain model of suicidal behavior in youth, if confirmed by future prospective studies, can have implications for clinical goals of early detection, risk stratification, and intervention development. Treatments that target emotional pain and social disconnect may be ideal interventions for reducing suicidality in adolescents.

## 1. Introduction

Alarmingly, rates of death by suicide have risen dramatically over the past two decades. The Centers for Disease Control and Prevention (CDC) reported a 30% increase in suicides in the United States from 2000 to 2016, with rates increasing among all age groups. Adolescents and young adults are at particularly high risk, concomitant with increased social media use, anxiety disorders, major depression, and self-inflicted injuries [1]. Between 2007 and 2017, the suicide rate in the U.S. nearly tripled for persons ages 10-14, increased by 76% for persons ages 15-19, and increased by 36% for persons ages 20-24 [2]. According to the most recent data from 2020, suicide is the second leading cause of death in persons aged 10-14 and 20-24 (surpassing homicide and second only to accidents) and the third leading cause of death in teens 15-19 years (with the rates of homicide being somewhat higher than those of suicide) [3].

Extensive research has identified risk factors for suicidal behavior, including chronic mental and physical illness, alcohol or drug abuse, acute emotional distress, and exposure to violence [4]. However, the predictive value of these factors remains limited, and reliable biomarkers for suicide risk have yet to be identified [5]. In order to develop targeted suicide prevention strategies and monitor patients' clinical response, objective biomarkers of suicide risk are urgently needed to address this escalating and important public health crisis.

Interest in the structural and functional neuroimaging correlates of suicidality as potential biomarkers has also grown over the past decade, though the majority of this research has focused on adult subjects. In adults, the most commonly reported findings center on the ventral and dorsal prefrontal cortices (vPFC and dPFC), the inferior frontal gyrus, the insula, and the mesial temporal, striatal, and posterior connections among these regions [6]. However, much less is known about the underlying neurobiology of suicidal ideation and attempts in adolescents [6]. Moreover, although some of this literature does focus on adolescents and young adult subjects, few authors have situated these neuroimaging findings in the context of the substantial neurodevelopmental changes that differentiate this population from adults [7]. Specifically, adolescence is the critical time of unique developmental sensitivity to social interactions [8]. Identifying biomarkers for screening and early detection among adolescents and preadolescents is critical, as these groups are particularly vulnerable to suicidal thoughts and behaviors, likely due to both psychosocial stressors that accompany the transition from childhood to young adulthood as well as underlying neurodevelopmental changes that occur during this period [9, 10]. Furthermore, treatments for major depressive disorder (such as agents targeting abnormal serotonergic signaling) have shown less efficacy for the treatment and prevention of suicidal ideation and suicide attempts, and treatments for acute suicidality (such as esketamine) are still in the early stages of investigation and appear to have a very limited duration of effect [11, 12]. Importantly, treatments for suicidality in adolescents significantly lag behind adults. Thus, there is an urgent need to fill the current gap in our neurobiological understanding of adolescent suicidal behavior in order to facilitate the development of effective acute and preventative clinical interventions.

A meaningful effort has been undertaken to develop a brain-centric model of suicidal behavior, one of the most comprehensive being the Mann and Rizk stress-diathesis model of suicidal behavior [13] (an update of the earlier neurobiological models of suicide presented in [14, 15]). The authors define diathesis as “a set of suicide-related traits that moderates the likelihood of suicidal behavior in response to stressors” [13]. The authors propose a model centered on four of such suicide-related and risk-moderating traits: (1) excessive subjective distress when depressed and attentional bias toward negative stimuli; (2) altered decision making with less delayed discounting and less executive control resulting in impulsive-aggressive tendencies and favoring acting on emotions; (3) neuropsychological abnormalities such as learning difficulties, cognitive rigidity, and memory problems; and (4) social distortions [13]. The authors suggest that the neurobiological correlates of these traits—such as neuroimaging signatures, peripheral inflammatory markers, cerebrospinal fluid (CSF) serotonin metabolite concentration, and cortisol levels—may serve as biomarkers for suicide risk [13]. Importantly, the identification of a neuroimaging signature of suicidal ideation could be a biomarker for suicide risk even when suicidal ideation (or related trait) is denied or unrecognized, since the biomarker is measured objectively. Moreover, such biomarker can be distinct from biomarkers of cooccurring psychiatric disorders [13]. Here, we build on this brain-centric model with a specific focus on adolescent neurocircuitry as described below.

In the present review, we summarize research on the neuroimaging correlates of suicidality in adolescents and young adults across structural and functional neuroimaging modalities. We summarize findings of 28 such studies. Because suicide is a transdiagnostic behavior, we put particular emphasis on convergent findings across studies and diagnostic groups. We also focus on findings in suicide attempters as they are the most clinically crucial category of patients for suicide prevention. To disentangle neural substrates of the mental disorder and suicidality, we focus on studies that compare the presence and absence of suicidal ideation and behavior *within* each diagnostic group. We then synthesize the findings and explore the functionality of the implicated brain regions and circuits. Finally, we propose a novel, neurodevelopmental model of suicidality in youth that borrows elements of Mann and Rizk stress-diathesis model of suicidal behavior and links them to the neuroimaging findings in adolescents.

## 2. Methods

To perform a systematic review of magnetic resonance imaging (MRI) literature that investigates neuroimaging correlates of suicidal ideation and behavior in adolescents, the electronic database PubMed was searched in March 2019 and updated in June 2021 using the following Boolean search term, applied to titles and abstracts: ((“MRI” [Title/Abstract] OR “DTI” [Title/Abstract] OR “magnetic resonance” [Title/Abstract]) AND (“suicidality” [Title/Abstract] OR “suicidal” [Title/Abstract] OR “suicide” [Title/Abstract]) AND (“adolescent” [Title/Abstract] OR “youth” [Title/Abstract])).

An additional updated search was conducted in May 2023, after the data synthesis had been performed and the brain model of suicidal behavior in youth had been built. The findings of this additional search are discussed in Discussion.

### 2.1. Data Synthesis

A high heterogeneity was expected among the studies with respect to the study population, type, and duration of training; studied brain regions; and, importantly, the specific MRI methodology. Therefore, a descriptive, *narrative synthesis* was chosen instead of a meta-analysis, as meta-analysis is not recommended for diverse study types [16]. The end goal of the synthesis was to summarize current research findings and derive a brain model of suicidal behavior in youth. Thus, we began with the systematic literature search first, then performed a narrative synthesis, and finally proposed a model.

## 3. Results

**Figure 1:**
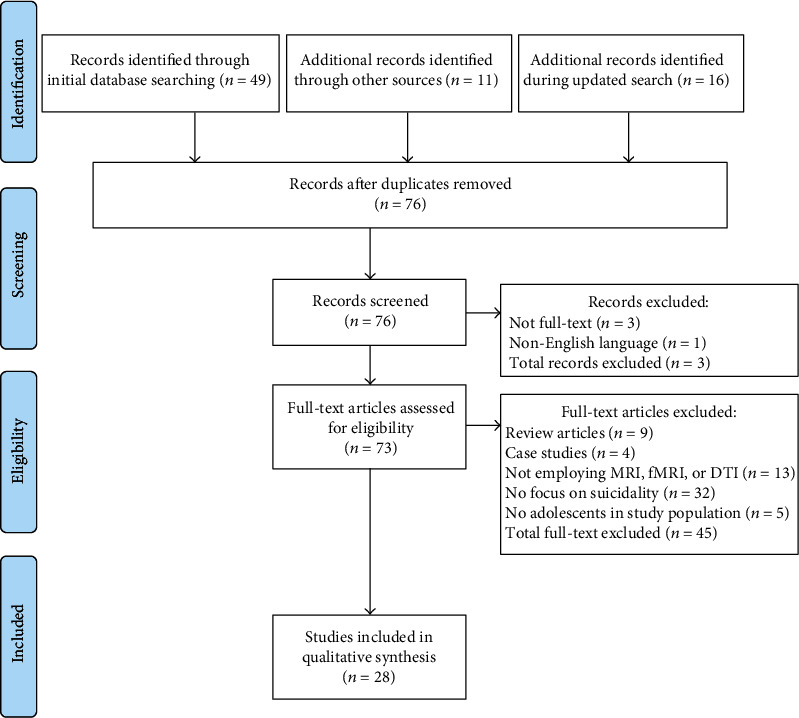
Literature search flow using PRISMA guidelines.

The initial search resulted in 49 entries (Figure 1). Eleven additional articles meeting the eligibility criteria were identified through other sources (citations in articles meeting the eligibility criteria). The updated search in June 2021 resulted in 16 additional records. After excluding articles that (a) were published in languages other than English (1 article), (b) were non-full-text articles (3), (c) were review articles (9), (d) were clinical case studies (4), (e) did not report neuroimaging findings (MRI, functional MRI (fMRI), or diffusion tensor imaging (DTI)) (13), (f) did not pertain to suicidal behavior or pertain to suicidal ideation or nonsuicidal self-injury alone (32), and (g) did not include adolescents in the study population (5), the total number of articles included in qualitative synthesis was 28. The search results and main findings of the included articles are summarized in Table 1.

Of the 28 included articles, all investigated neuroimaging correlates of suicidality in clinical populations. Twelve of the articles investigated subjects with unipolar depression and healthy controls; nine articles studied clinically mixed populations containing subjects with bipolar disorder, unipolar depression, and other psychiatric diagnoses (proportions reported under “subject clinical status” in Table 1 for these studies); five articles studied only subjects with unipolar depression, one of which studied only subjects with treatment-resistant unipolar depression; one article studied subjects with bipolar disorder and healthy controls; and one article studied only subjects with bipolar disorder (Table 1).

To better categorize the included studies, we created a 2 × 2 table (Table 2) that separates the study findings by diagnosis (major depressive disorder (MDD) vs. other conditions or heterogeneous groups) and suicidal ideation (SI) vs. suicide attempt (SA). We defined suicidal ideation as thinking about, considering, or planning suicide [17]. Study results relevant to individuals who fit that definition, as stated by study authors, were delineated into the category of SI. We defined suicide attempt as a potentially self-injurious act committed with at least some wish to die [18]. Study results relevant to individuals who fit that definition, as stated by study authors, were delineated into the category of SA. This resulted in the following four categories: MDD+SI, MDD+SA, heterogeneous+SI, and heterogeneous+SA (Table 2). In this descriptive approach, we indicate next to each brain region the frequency with which this region was reported as a finding. While a very large number of regions are listed in Table 2, we focused on convergent findings across studies and diagnostic groups, since suicide is a transdiagnostic behavior, and on findings in attempters specifically as the most crucial category of patients for suicide prevention (categories MDD+SA and heterogeneous+SA in Table 2). At the same time, *within* each study, we focused on comparisons of the presence and absence of suicidal ideation and behavior *within* a diagnostic group, to disentangle neural substrates of the mental disorder and suicidality.

Given the described focus, the most common transdiagnostic findings in suicide-attempting patients compared to nonattempting or healthy controls included abnormalities of the cerebellum, hippocampus, amygdala, lateral orbitofrontal cortex (OFC), temporal gyrus, and connections between the latter two (Table 2). Details of the findings related to these brain regions are provided below.

### 3.1. Cerebellum Findings

Four of the reviewed studies identified abnormalities of the cerebellum in subjects with a history of suicide attempt. Adolescents with bipolar disorder and a history of suicide attempt showed reduced gray matter volume of the bilateral cerebellum and reduced white matter fractional anisotropy (FA) in the right cerebellum compared to those without a history of suicide attempt [19]. In a facial recognition task, adolescents with high suicidality (often attempters) and MDD demonstrated increased activation in the left anterior lobe of the cerebellum [20]. Observed abnormalities in resting-state functional connectivity included increased resting-state functional connectivity (rs-fc) between the right precuneus and bilateral cerebellum and decreased rs-fc between the left cerebellum and the left posterior cingulate cortex, lateral occipital cortex, and temporal-occipital fusiform gyrus [21] and increased rs-fc in the left cerebellum [22].

### 3.2. Hippocampus Findings

Two of the reviewed studies identified abnormalities of the hippocampus in subjects with a history of suicide attempt. Among adolescents with bipolar disorder, Johnston et al. reported reduced right hippocampal gray matter volume in those with a history of suicide attempt compared to those without [19]. Among adolescents with major depressive disorder, Quevedo et al. reported reduced activity of the left and right hippocampi during an emotional facial self-recognition task in those with high suicidality compared to those without [20].

### 3.3. Amygdala Findings

Three of the reviewed studies identified functional abnormalities of the amygdala in adolescents with a history of suicide attempt, each of which examined clinical populations using task-based fMRI. In a study comparing subjects with MDD with prior suicide attempts, subjects with MDD and suicidal ideation symptoms but no prior suicide attempts, and healthy controls during a facial emotion processing task, Alarcón et al. found that those with prior suicide attempts had greater connectivity between the amygdala and the dlPFC, dmPFC, precuneus, and ACC than low-suicidality depressed subjects, as well greater connectivity between the left amygdala and the rostral anterior cingulate cortex (rACC) than all other groups [23]. In a study using a similar facial emotion processing task limited to subjects with bipolar disorder, Johnston et al. report reduced amygdala functional connectivity to the left ventral PFC and right rostral PFC in subjects with a history of a suicide attempt compared to those without prior attempts [19]. In another study of subjects with unipolar depression, Quevedo et al. found reduced activity in the amygdala in response to happy faces during a facial self-recognition task [20].

### 3.4. Lateral OFC Findings

Three of the reviewed studies identified abnormalities of the lateral OFC in subjects with a history of suicide attempt. In a study of subjects with MDD, those with a history of suicide attempt demonstrated reductions in the right and left lateral OFC thickness and pars orbitalis thickness relative to the non-suicide-attempting subjects [24]. Additionally, subjects with bipolar disorder and a history of suicide attempt showed reduced left and right lateral OFC thickness relative to those without a history of suicide attempt [25]. Similarly, Johnston et al. [19] report reduced right lateral OFC volume in subjects with a history of bipolar disorder and suicide attempt compared to peers without a history of suicide attempt.

### 3.5. Temporal Gyrus Findings

*Middle temporal gyrus*: two of the reviewed studies identified abnormalities of the middle temporal gyrus (MTG) in subjects with MDD and a history of suicide attempt, specifically increased activity in the right MTG in response to angry faces in a facial emotion processing task [26] and increased resting-state amplitude of low-frequency fluctuations in the left MTG [27]. *Superior temporal gyrus*: three of the reviewed studies identified abnormalities of the superior temporal gyrus (STG) in subjects with MDD and a history of suicide attempt, including reduced temporal pole volume [24], reduced right STG volume [28], and increased resting-state amplitude of low-frequency fluctuations in the right STG [27].

### 3.6. Connections between Lateral OFC and Temporal Gyrus

Two of the reviewed studies identified abnormalities of the uncinate fasciculus in the heterogeneous group with a history of suicide attempt, including reduced fractional anisotropy in the left frontotemporal white matter, including the uncinate fasciculus [29], and reduced fractional anisotropy in the uncinate fasciculus and the left uncinate-vPFC region [19]. Fan et al. additionally identified reduced fractional anisotropy in the uncinate fasciculus in subjects with MDD and a history of suicide attempt [29]. Since the uncinate fasciculus is a white matter association tract that connects the temporal pole with the OFC, we consider the findings of reduced temporal pole [24] and OFC volume [24] in MDD subjects and a history of suicide attempt to be an additional indication of the frontotemporal circuit aberration.

The following list summarizes which cross-diagnostic neural aberrations listed above were observed in suicide attempters (compared to nonattempting ideators) and in nonattempting ideators (compared to nonideators), how many studies reported such aberrations, and what type of MRI was utilized.

(i) *Cerebellum findings*
  - Suicidal ideation was associated with decreased cerebellum activity (2 studies) and inconsistent changes in resting-state functional connectivity with other regions (2 studies)
  - Suicide attempt was associated with reduced cerebellum gray matter volume (1) and increased connectivity between the cerebellum and the left lingual gyrus (1)
(ii) *Hippocampus findings*
  - Suicidal ideation was associated with reduced hippocampus activity (2)
  - Suicide attempt was associated with reduced hippocampus volume (1)
(iii) *Amygdala findings*
  - Suicidal ideation was associated with reduced activity in the amygdala (1) and greater connectivity between the amygdala and dlPFC (1) and the amygdala and rACC (1)
  - Suicide attempt was associated with greater connectivity between the amygdala and dlPFC (1) and the amygdala and rACC (1) and reduced amygdala connectivity to the ventral PFC (1)
(iv) *Lateral OFC findings*
  - Suicide attempt was associated with decreased lateral OFC thickness (5) and decreased OFC volume (1)
(v) *Temporal gyrus findings*
  - Suicide attempt was associated with decreased temporal pole volume (1), decreased right superior temporal gyrus volume (1), increased temporal gyrus activity (3), and reduced fractional anisotropy in temporal regions (3)
(vi) *Connections between lateral OFC and temporal gyrus*
  - Suicide attempt was associated with reduced fractional anisotropy in the uncinate fasciculus (3)

### 3.7. Summary of the Main Findings

The most common transdiagnostic abnormalities in suicide-attempting adolescent patients compared to nonattempting or healthy controls were found in cerebellum, hippocampus, amygdala, lateral orbitofrontal cortex (OFC), temporal gyrus, and connections between the latter two, whereas abnormalities in the cerebellum, hippocampus, and amygdala were also present in nonattempting ideators (probably to a lesser degree); lateral OFC and temporal circuitry abnormalities appear to be unique to adolescent suicide attempters.

We suggest that abnormalities in the cerebellum, hippocampus, and amygdala may be present in nonattempting ideators to a lesser degree compared to attempters based on the following consideration. If a brain region appears in both columns of Table 2, it means that this brain region showed different properties in comparison between ideators and nonideators, as well as between suicide-attempting subjects compared to nonattempting subjects from the same clinical group (and thus likely ideators). Assuming a monotonic property, we therefore hypothesize that the most likely explanation is that while the aberration in this brain region is present both in ideators and in attempters, it is monotonically stronger in attempters (otherwise, there would have been no findings in the comparison between suicide-attempting subjects and nonattempting subjects from the same clinical group).

## 4. Discussion

**Figure 2:**
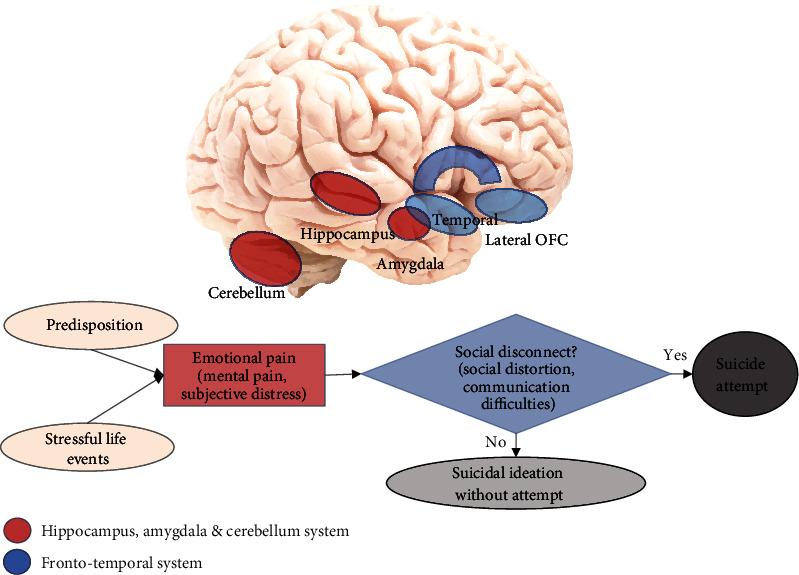
Emotional paiN and social Disconnect (END) brain model of suicidal behavior in youth. Emotional/mental pain or subjective distress can be caused by a combination of predisposition and stressful life events and leads to suicidal ideation in adolescents. If, in addition, the adolescent is experiencing social disconnect/distortion and communication difficulties, this can lead to a suicide attempt. According to the END model, the biological driver of suicidal behavior in youth is aberration in two distinct brain circuits: “emotional pain” circuit and “social disconnect” circuit. The emotional pain circuit includes the cerebellum, hippocampus, and amygdala and shows similar aberrations in adolescent ideators as in attempters, but to a smaller degree. The social disconnect circuit includes lateral OFC and temporal gyri, as well as the connections between them (the frontotemporal system), and is unique to adolescent suicide attempters. Abbreviation: OFC = orbitofrontal cortex.

Our systematic review highlighted several brain regions/connections that may be associated with adolescent suicide attempts across psychiatric diagnoses. We suggest that these findings can be subdivided into two circuits that roughly correspond to traits 1 and 4 in the Mann and Rizk stress-diathesis model of suicidal behavior described in the introduction [13]: (1) “emotional pain” circuit and (2) “social disconnect” circuit (Figure 2). The proposed emotional pain circuit includes the cerebellum, hippocampus, and amygdala, whereas the social disconnect circuit includes the lateral OFC and temporal gyrus, as well as connections between the two. Below, we discuss these findings and propose a new brain model of suicidal behavior in youth based on the synthesis of these findings: Emotional paiN and social Disconnect (END) model (Figure 2).

### 4.1. Emotional Pain

Emotional pain, also referred to as mental pain, psychological pain, or subjective distress, is thought to be a key component in understanding suicidal thoughts and behaviors [30]. Higher levels of emotional pain are associated with a higher risk for suicide [31]. Specifically, those with suicidal thoughts report higher levels of emotional pain than those without these thoughts, and those with a history of suicidal behavior report higher levels of emotional pain than those who do not have a history of suicidal behavior [31]. Furthermore, these results persist when analyzed among groups with similar levels of depression, suggesting that the association between emotional pain and suicidality is independent of depressive symptoms [32]. Emotional pain *tolerance* may be an even better indicator for suicidal behavior [33].

The relationship between emotional pain and suicidality provides an important insight because it suggests that suicidal thoughts are utilized as a way to cope with such pain. When one experiences distress, and subsequently emotional pain, suicide may be considered as a way to escape this feeling. In the early 1990s, Shneidman et al. proposed a theory of suicide in which emotional pain, for this reason, was a necessary phenomenon in the etiological development of suicidal thoughts [30]. This idea was then corroborated by the escape theory of suicide, which hypothesized the same idea that suicidal thoughts are utilized in order to escape mental anguish [34]. More recently, suicidologists have built on this idea. Kleiman et al. [35] found that some individuals may experience improved mood as a result of suicidal thoughts. This finding suggested that suicidal thoughts may act as a reinforcing mechanism, increasing their presence and frequency in response to subjective distress.

It is well established that the endocannabinoid system plays an important modulatory role in processing of both physical pain [36, 37] and emotional/social pain [38]. We suggest that the emotional pain associated with adolescent suicidality is (at least in part) facilitated by three brain regions derived from our review, in which endocannabinoid signaling has been shown to play important roles: the hippocampus, amygdala, and cerebellum [39]. Indeed, it has been suggested that the endocannabinoid system, and particularly the cannabinoid receptors CB1, may be involved in the pathogenesis of suicidal behavior in adult patients with different affective disorders and alcoholism [40, 41]. Out of the three brain regions, the cerebellum received the greatest and most consistent support for being associated with emotional pain [42]. Among the studies reviewed here, aberrant cerebellum activity during emotion processing tasks has been linked to adolescent suicidal ideation (e.g., [43]) and attempts [19–22]. Interestingly, an association between increased metabolism in the cerebellum and reductions in suicidal ideation has been previously observed following treatment with ketamine in adults [44]. Although not classically associated with the *experience* of emotional pain, the amygdala and hippocampus appear to play a modulatory role. Whereas the studies reviewed here link them with adolescent suicidal ideation and attempts (e.g., [19, 20, 23]), differences in characteristics of these brain regions have been suggested in general to predispose subjects to chronic pain and mood disorders [45]. Moreover, amygdala circuitry plays a key role during cannabinoid analgesia in animals [46] and humans [47], and amygdala connectivity correlates with the dissociative effects of cannabis administration on the reported intensity and *unpleasantness* of pain [47]. It is also interesting to note that in adults diagnosed with complicated grief (i.e., grief strongly associated with emotional pain and subsequently suicidality [48]), all three regions—the hippocampus, amygdala, and cerebellum—showed interactive activation differences in response to positive/negative valence images compared to controls [49]. The interactions were driven by (1) the greater activation of the amygdalae, cerebellum, and right hippocampus in the grief group while viewing death-related pictures and (2) the greater deactivation of these regions in the same group while viewing positive valence pictures.

To conclude, the hippocampus-amygdala-cerebellar network's aberrations may be associated with the emotional pain experienced by adolescents with suicidal thoughts and behaviors. The observation of such aberrations both in attempters and ideators (albeit likely to a lesser degree in the latter) suggests that these aberrations and the associated emotional pain may be a necessary, but not sufficient condition, for attempting suicide by the adolescent.

### 4.2. Social Disconnect

Social distortion/disconnect, “thwarted belongingness” (a perceived lack of reciprocally caring relationships [50]), and communication difficulties have been suggested to increase suicide risk [13]. Some authors have even suggested that social disconnect is a second necessary component, in addition to emotional pain, which leads to suicide attempts [51]. For example, Levi et al. observed that while emotional pain predicted the presence of suicidal ideation and medically nonserious suicidal behavior in adults, “unrelieved” emotional pain due to communication difficulties (e.g., difficulties with self-disclosure and inability to ask for help) predicted the lethality and seriousness of the suicide attempts [51].

The most comprehensive description of how social variables may be impacting suicidality is given by Thomas Joiner's interpersonal theory of suicide (IPTS) [50, 52]. The IPTS proposes that a person's suicidality is associated with their perception of their interpersonal relationships. According to the IPTS, the desire to die is the result of two constructs: thwarted belongingness and perceived burdensomeness. Thwarted belongingness refers to a feeling of isolation, loneliness, or the lack of reciprocal support by others. Perceived burdensomeness represents one's feeling of being a burden to those around them, or a feeling that others may be better off without them. The IPTS posits that when these constructs appear simultaneously, one is at risk for suicidal thoughts. Furthermore, when these thoughts occur simultaneously with the acquired capability of suicide that counteracts the evolutionary fear of death and physical pain, one is at risk for a lethal suicide attempt [50].

While IPTS found preliminary empirical support in adolescents [53], it has been suggested that the interpersonal factors contributing to suicidal ideation (e.g., thwarted belongingness and perceived burdensomeness) are consistently enmeshed in adolescence and may be better conceptualized as a single composite construct [53]. For this reason, and because differentiating social variables on the level of large-scale brain networks might be especially difficult, we combine these variables and conceptualize them as “social disconnect” in our brain model of suicidal behavior in youth described below. Similarly, Mann and Rizk's brain-centric stress-diathesis model of suicidal behavior only includes “social distortion” as a social risk-moderating trait [13].

According to Mann and Rizk's model, the social distortion trait is associated with a brain region also found in our review, the lateral OFC, and its hyperactivity to negative facial expressions and hypoactivity to positive facial expressions [13]. In our reviewed literature, in a study of subjects with MDD, those with a history of suicide attempt demonstrated reductions in right and left lateral OFC thickness and pars orbitalis thickness relative to the non-suicide-attempting subjects [24]. Additionally, subjects with bipolar disorder and a history of suicide attempt showed reduced left and right lateral OFC thickness relative to those without a history of suicide attempt [25]. Johnston et al. [19] reported reduced right lateral OFC volume in adolescent subjects with a history of bipolar disorder and suicide attempt compared to peers without a history of suicide attempt. This result is aligned with adult neuroimaging studies demonstrating associations between attempt lethality and ventral prefrontal hypometabolism [54]. Interestingly, based on lethality's association with highly planned, less impulsive attempts [54] and the lack of associations between impulsivity and reductions in structural and functional neural integrity among attempters, Johnston et al. suggested that “altered ventral prefrontal functioning in attempt lethality may not, at least for some individuals, be related to its role in controlling impulses” [19] In addition to the OFC (and amygdala), the temporal gyrus is known to be one of the three major components of the “social brain” [55], and multiple papers reviewed here showed temporal gyrus aberrations in adolescent subjects with a history of suicide attempt [24, 26–28]. Finally, perturbations of the uncinate fasciculus that connects the lateral OFC and temporal gyrus may contribute to adolescent suicidal behavior by causing social-emotional problems, such as distortion of personal value and emotional history associated with people, disruption of the social reward mechanism, lack of social engagement, and potentially antisocial behavior [56]. Vulnerability to such social-emotional problems may be especially high during adolescence because the uncinate fasciculus continues to mature during this important neurodevelopmental period [57].

To conclude, the frontotemporal network aberrations may be associated with the social disconnect experienced by adolescents with increased suicidality. The observation of this network aberrations appears to be unique to adolescents with a history of a suicide attempt, compared to nonattempting ideators.

### 4.3. END Model

We synthesize the findings described above into the Emotional paiN and social Disconnect (END) brain model of suicidal behavior in youth (Figure 2). In this model, emotional/mental pain or subjective distress can be caused by a combination of predisposition and stressful life events and leads to suicidal ideation in adolescents. If, in addition, the adolescent is experiencing social disconnect/distortion and communication difficulties, this combination can lead to a suicide attempt. According to the END model, the biological driver of suicidal behavior in youth is aberrations in two distinct brain circuits: the “emotional pain” circuit and the “social disconnect” circuit. The emotional pain circuit includes the cerebellum, hippocampus, and amygdala and shows similar aberrations in adolescents with suicidal ideation as in those with a history of a suicide attempt, but to a smaller degree. The social disconnect circuit includes the lateral OFC and temporal gyri, as well as the connections between them (i.e., the frontotemporal system), and is unique to adolescent suicide attempters, compared to nonattempting ideators.

As mentioned in Methods, we conducted an additional updated search in May 2023, after the data synthesis had been performed and the brain model of suicidal behavior in youth had been built. The search resulted in 8 papers that met the inclusion criteria [58–65]. To preserve the order of the data synthesis—in which we begin with the systematic literature search first, without any specific model in mind, then perform a narrative synthesis, and finally propose a model—we did not include the 8 additional papers in the synthesis and instead compared the findings in these papers to our END model as to whether they support or contradict it. Given that our END model focused on cross-diagnostic suicidal behavior, we focused on the studies that reported nonnegative findings in heterogeneous adolescent populations with a direct measurement of suicide attempts. The two studies that met this definition showed a general agreement with the END model, highlighting frontotemporal structural and right OFC functional aberrations in youth with bipolar disorder and self-harm [60, 61]. The largest study among the 8 additional studies was the ENIGMA Suicidal Thoughts and Behaviours (ENIGMA-STB) consortium study (comprised of multiple samples, including a sample of 577 young people with mood disorders and a transdiagnostic sample with 253 healthy controls, 432 clinical controls, and 91 young people with a history of a suicide attempt), which did not show any morphological findings in the heterogeneous cohort of young people with a history of suicide attempts but showed a correlation of suicide attempt history with a smaller surface area of the frontal pole in the mood disorder cohort [65]. Even though the absence of findings in the heterogeneous cohort makes this study not directly comparable with the END model, it is worth mentioning that the frontal pole is adjacent to the lateral OFC, which may point to compatibility with the model.

### 4.4. Comparison with Adult Literature

While the END model has been derived from adolescent literature, it may, in principle, also apply to adults. At the same time, there may be some unique aspects that characterize the adolescent brain, which we would like to discuss here. Notably, the adult literature reports impairments in vPFC and dPFC and decreased top-down inhibition of behavior that is associated with suicidal behavior [6]. The literature in youth reviewed here does not provide strong support for abnormalities in PFC regions associated with top-down inhibition and planning. Rather, we observe a strong support for a unique role of the lateral OFC, which is unlikely related to controlling impulses [19]. As mentioned above, Johnston et al. suggested that aberrations in OFC's functioning in attempt lethality may not be related to its role in controlling impulses [19]. Instead, it is likely that in combination with the temporal regions, the lateral OFC constitutes a network that may be associated with social disconnect (Figure 2). Adolescence is a critical time of unique developmental sensitivity to social interactions [8]; therefore, aberrations in the underlying circuitry may reflect the lack of the social connectedness that may otherwise reduce the likelihood of a suicide attempt in a person experiencing severe emotional pain.

### 4.5. Clinical Implications

The convergent findings from the reviewed literature and our proposed END brain model of suicidal behavior in youth, if confirmed by future prospective studies, can have implications for eventual clinical goals of early detection, risk stratification, and intervention development. Our model suggests that future research should consider therapies such as interpersonal psychotherapy for depressed adolescents (IPT-A) for reducing suicidality or new interventions that would address the social disconnect [66, 67]. An effective intervention for reducing social disconnect could also open a way to the treatment of emotional pain [51]. For example, “TARA”—an innovative NIH Research Domain Criteria (RDoC) and neuroscience based intervention for adolescent depression—targets, among other brain regions, the hyperactive amygdala and may serve as an example of neurocircuitry-driven intervention development [68–70].

### 4.6. Limitations

There are two main limitations to this review: a large variability of clinical diagnoses and MRI methodology in the included studies. The differences in MRI methodology precluded us from conducting a meta-analysis; thus, a descriptive assessment of the frequency with which certain findings are reported was used instead. Since findings depend on the MRI methodology, our synthesis may be biased in favor of findings that are easily detected by the most frequently used MRI methodologies. It is reassuring, however, that there was a noticeable convergence of findings obtained with highly varying methodologies (e.g., volumetry vs. task-based fMRI). It is worth noting that 25% of all included papers derived their results from resting-state functional connectivity. While our END model, to a large extent, focuses on discrete brain regions, future work may benefit from network science approaches to neuroimaging of suicidality [71]. To address the second issue of varying clinical diagnoses, we focused (1) only on comparing findings in attempters vs. nonattempters *within* the diagnostic category and (2) on the brain circuitry aberration across all diagnoses when synthesizing the findings. Finally, our proposed END brain model of suicidal behavior in youth is simplified in that it focuses only on the most general mechanisms supported by the largest number of findings, leaving out several other implicated brain regions and circuits (Table 2). While this simplification does not allow us to capture the full complexity of the phenomenon of suicidality, the model highlights the mechanisms most likely to be dominant and thus those which may best facilitate the development of practical, targeted clinical interventions.

## 5. Conclusions

In this systematic review, we descriptively synthesized results from 28 studies on the neural underpinnings of suicidal behavior in adolescents that used various MRI methods. The brain regions that were the most common transdiagnostic findings in suicide-attempting patients compared to nonattempting or healthy controls were cerebellum, hippocampus, amygdala, lateral orbitofrontal cortex (OFC), temporal gyrus, and connections between the latter two. We proposed the Emotional paiN and social Disconnect (END) model of adolescent suicidality, according to which suicidal behavior is driven by two key neural circuits: (1) emotional/mental pain circuit and 2) social disconnect/distortion circuit. In the END model, the emotional pain circuit, consisting of the cerebellum, amygdala, and hippocampus, shows similar aberrations in adolescents with suicidal ideation as in those with a history of a suicide attempt (but to a smaller degree). The social disconnect circuit is unique to adolescent suicide attempters and includes the lateral OFC, the temporal gyri, and the connections between them. Using the END model, we discussed potential clinical treatments and suggested that future research should consider therapies such as IPT-A for reducing suicidality or new interventions that would address the social disconnect.

## Figures and Tables

**Table 1:** Summary of the included articles.

Diagnosis	First author (publication year)	Number of subjects	Subject age in years (range or mean ± SD)	Subject sex	Subject clinical status	Comparison groups	Measure of suicidality	MRI method(s)	Significant MRI findings
Suicidal ideation									
MDD	Cullen [72]	70	12–19 Mean not reported	Male (23%) and female (77%)	Unipolar depression, healthy controls	MDD vs. healthy controls	Inventory of Depression and Anxiety Symptoms (IDAS), Kiddie Schedule for Affective Disorders and Schizophrenia, Children's Depression Rating Scale-Revised	Resting-state fMRI	Adolescents with MDD showed lower positive resting-state functional connectivity (RSFC) between the amygdala and hippocampus, parahippocampus, and brainstem (*z* > 2.3, corrected *p* < 0.05). Patients also demonstrated greater (positive) amygdala-precuneus RSFC (*z* > 2.3, corrected *p* < 0.05) in contrast to negative amygdala-precuneus RSFC in the adolescents serving as controls.
	Ordaz [73]	40	14–17 16.15 ± 1.12	Male (25%) and female (75%)	Unipolar depression	MDD	Columbia Suicide Severity Rating Scale	Resting-state fMRI	Lower coherence in the left executive control network (Δ*R*^2^ = 0.264), anterior default mode network (Δ*R*^2^ = 0.118), and salience network (Δ*R*^2^ = 0.164) was independently associated with greater lifetime severity of suicidal ideation.
	Quevedo [20]	119	11–18 14.79 ± 1.64	Male (39%) and female (61%)	Unipolar depression, healthy controls	MDD+low suicidality vs. MDD+high suicidality vs. healthy controls	Child Depression Rating Scale (CDRS), Moods and Feelings Questionnaire (MFQ)	Task-based fMRI (facial self-recognition task)	High-suicidality (HS) depressed subjects showed greater activity in the bilateral cuneus and occipital gyrus versus both low-suicidality (LS) depressed subjects and healthy controls (HC) (*F* = 23.34). HS and LS subjects showed greater activity in the right inferior frontal gyrus than HC subjects (*F* = 14.41). A group by self by emotion interaction with HS showed lower activity in the right hippocampus (*F* = 5.28), the left hippocampus and amygdala (*F* = 4.67), and the medial prefrontal cortex (mPFC) and Brodmann's area (BA) 10 (*F* = 4.76) in the happy self versus other face condition relative to the LS group, who had less activity in these regions compared to HC subjects. In a subsequent analysis that included depression severity as a covariate, a group by self-interaction showed that LS demonstrated higher midline cortical structure (MCS) activity than HS for the self versus other faces including in the left anterior lobe of the cerebellum and culmen (*F* = 11.49); the bilateral precuneus, posterior cingulate, and BA31 (*F* = 9.21); and the bilateral anterior cingulate, mPFC, BA32, and BA10 (*F* = 9.07).
	Schreiner [21]	58	12–19 15.78 ± 1.86	Male (22%) and female (78%)	Unipolar depression with various comorbid conditions (40% GAD, 16% ADHD, and 12% eating disorder NOS)	MDD (within group)	Inventory of Depression and Anxiety Symptoms (IDAS)	Resting-state fMRI	IDAS suicidality score was positively correlated with resting-state functional connectivity (RSFC) between the left precuneus and left pre- and postcentral gyri, superior and middle frontal gyri, and superior parietal lobe (*z*-score range 3.10-3.56 for entire cluster). IDAS suicidality score was also positively correlated with RSFC between the right precuneus and the left pre- and postcentral gyri (*z*-score range 3.22-3.62 for entire cluster), bilateral cerebellum (*z*-score range 3.02-4.06 for entire cluster), and right precentral gyrus and middle, inferior, and superior frontal gyri (*z*-score range 2.81-3.70 for entire cluster). IDAS suicidality score was negatively correlated with RSFC between the left posterior cingulate cortex and left cerebellum, lateral occipital cortex, and temporal-occipital fusiform gyrus (*z*-score range 3.37-3.96 for entire cluster).
	Schwartz [74]	33	Baseline: 16.33 ± 1.03; follow-up: 16.80 ± 1.06	Male (24%) and female (76%)	Unipolar depression	MDD (within group)	Columbia Suicide Severity Rating Scale	Resting-state fMRI	In this longitudinal study, the degree of reductions in clinical measures of subject lifetime suicidal ideation was correlated with the degree of increase in resting-state salience network (SN) coherence (*β* = −0.50, *t* = −3.05).
Heterogeneous	Dir [75]	57	11-12 SI: 11.88 ± 0.48 Non-SI: 11.91 ± 0.53	Male (63%) and female (37%)	ADHD (43.9%), disruptive behavior disorders (64.9%)	Heterogeneous+SI vs. heterogeneous-SI	Schedule for Affective Disorders and Schizophrenia for School-Aged Children (K-SADS-PL)	Task-based fMRI (balloon analog risk task), reconstructed sMRI	Subjects showed relative increase in frontoparietal activation when choosing the win option as explosion probability increased in balloon analog risk task. Subjects with SI showed increased activity in the left precentral gyrus when choosing the win option and decreased activity when choosing the inflate option as explosion probability increased. Subjects without SI showed similar activity regardless of explosion probability. As explosion probability increased, subjects with SI showed greater orbitofrontal cortex activation to inflations, while subjects without SI had greater orbitofrontal cortex activation during explosions.
	Ho [76]	152	11.41 ± 1.01	Male (41%) and female (59%)	Axis I disorders not specified (20.4%), healthy subjects	Heterogeneous	Suicidal Ideation Questionnaire (SIQ)-Junior High Version; Implicit Association Task (IAT)-Death Version	sMRI	Reduced gray matter volume (GMV) of the bilateral putamen was significantly associated with higher IAT *d*-scores (left: *t* = 2.597; right: *t* = −3.374). Reduced left caudate GMV was also significantly associated with higher IAT *d*-scores (*t* = 2.597).
	Miller [43]	49	13–20 16.95 ± 1.54	Male (41%) and female (59%)	Mixed: proportions not reported	Heterogeneous+SI vs. heterogeneous-SI	Composite score: Scale for Suicidal Ideation, Diagnostic Interview Schedule for Children Version IV (DISC-IV), Self-Injurious Thoughts and Behaviors Interview	Task-based fMRI (emotion regulation task)	Subjects with suicidal ideation (SI) demonstrated greater activation of the right dorsolateral prefrontal cortex (dlPFC) compared to subjects without SI (*z* = 3.71) on trials in which they attempted to regulate their emotional responses to stimuli compared with trials in which they passively viewed negative emotional stimuli. During passive viewing of negative emotional stimuli, subjects with SI demonstrated less activation in the right dlPFC (*z* = 4.03), right temporoparietal junction (*z* = 3.94), right thalamus (*z* = 3.99), and left cerebellum/lateral occipital lobe (*z* = 3.67) than age-matched controls and greater activation in the left temporal pole (*z* = 3.48) than age-matched controls.
	Oppenheimer [77]	36	13.56 ± 1.50	Male (47%) and female (53%)	Mixed: anxiety disorders (28%)	Heterogeneous	Schedule for Affective Disorders and Schizophrenia for School-Aged Children (K-SADS); SI Composite of the Mood and Feelings Questionnaire (MFQ-SI41); Children's Depression Rating Scale-Revised (CDRS-R)	Task-based fMRI	Subjects with high levels of peer victimization or ecological momentary assessment-measured daily negative social experiences showed positive association between right anterior insula activation and suicidal ideation.
	Vidal-Ribas [78]	7994	9-10 Mean not reported	Male (53%) and female (47%)	Depression, anxiety disorders, ADHD, oppositional defiant disorder, conduct disorder, PTSD	Heterogeneous+SI vs. heterogeneous-SI	Schedule for Affective Disorders and Schizophrenia for School-Aged Children (K-SADS-PL)	Resting-state fMRI, sMRI	Subjects with caregiver-reported suicidal thoughts and behaviors had thinner superior temporal sulcus left banks when compared with the never-suicidal group (*b* = 20.04, *t* = 22.93, df = 5040, *p* = 0.003). Subjects with a history of suicide attempts exhibited greater activation in the pallidum and decreased activation in the ventral diencephalon during inhibitory control tasks.

Suicide attempt									
MDD	Alarcón [23]	120	11–17 HC: 14.46 ± 1.52 LS: 14.87 ± 1.75 HS: 15.04 ± 1.68 SA: 14.61 ± 1.57	Male (46%) and female (54%)	Unipolar depression, healthy controls	MDD+low SI vs. MDD+high SI vs. MDD+SA vs. healthy controls	Schedule for Affective Disorders and Schizophrenia for School-Aged Children (K-SADS-PL), Child Depression Rating Scale	Task-based fMRI (facial emotion processing task)	High-suicidality (HS) and suicide-attempting (SA) depressed subjects showed greater connectivity between the amygdala and the dorsolateral prefrontal cortex (dlPFC) (LS: *t* = 4.28, HS: *t* = 4.60, SA: *t* = 4.41), dorsomedial prefrontal cortex (dmPFC) (LS: *t* = 3.61, HS: *t* = 4.08, SA: *t* = 4.47), and precuneus (LS: *t* = 3.95, HS: *t* = 4.32, SA: *t* = 4.44) than low-suicidality (LS) depressed subjects, who demonstrated a greater degree of amygdala-dlPFC connectivity than healthy controls (HC). Suicide-attempting depressed subjects showed greater connectivity between the left amygdala and the rostral anterior cingulate cortex (rACC) (HC<LS: *t* = 4.30; HC<HS: *t* = 3.52: HC<SA, *t* = 3.08) than all other groups. HS showed greater right amygdala-rACC connectivity than LS (*t* = 3.80) and HC (*t* = 3.46).
	Cao [27]	100	15–29 Subgroup means SU: 20.63 ± 3.65 NSU: 21.39 ± 3.05 HC: 20.53 ± 1.84	Male (34%) and female (66%)	Unipolar depression, healthy controls	MDD+SA vs. MDD-SA vs. healthy controls	History of prior attempt by clinical interview	Resting-state fMRI	Compared with nonsuicidal subjects and healthy controls, the suicide-attempting group exhibited increased zALFF (*z*-score fractional amplitude of low-frequency fluctuation) in the right superior temporal gyrus (*t* = 2.38, 4.23, respectively), left middle temporal gyrus (*t* = 3.99, 4.46, respectively), and left middle occipital gyrus (*t* = 4.09, 3.09, respectively). Significantly decreased zALFF was observed in the suicidal group in the left superior frontal gyrus (*t* = 4.20) and left middle frontal gyrus (*t* = 4.20) compared to the nonsuicidal group.
	Ehrlich [79]	102	26.7 ± 5.5	Male (33%) and female (67%)	Unipolar depression	MDD+SA vs. MDD-SA	History of prior attempt by clinical chart review	sMRI	History of suicide attempt(s) was significantly correlated with the presence of periventricular white matter hyperintensities (PVHs) (Fisher's exact test: *p* = 0.02), as well as right hemisphere PVHs (Fisher's exact test: *p* = 0.04). Logistic regression analyses controlling for age, sex, and several clinical risk factors supported this finding (OR = 5.7; 95% CI: 1.6-21.2).
	Fradkin [80]	58	12–19 Control: 16.9 ± 1.718 MDD: 17.6 ± 1.821	Male (21%) and female (79%)	Unipolar depression with a history of at least one suicide attempt (50%); healthy controls (50%)	MDD+SA+high impulsivity vs. MDD+SA+low impulsivity vs. healthy controls	Reynolds' Suicidal Ideation Questionnaire (SIQ and SIQ-JR); Suicide Intent Scale (SIS)	sMRI	While healthy controls demonstrated a positive association between cortical thickness in the anterior left rostral middle frontal region (vmPFC) and BIS-11 motor impulsivity scores, participants with MDD and a history of at least one suicide attempt demonstrated a negative association between vmPFC cortical thickness and BIS-11 motor impulsivity scores (Cohen's *d* = 0.42, *p* = 0.02).
	Hong [24]	66	14-25 SA: 19.54 ± 2.86 SI: 18.38 ± 3.94	Male (30%) and female (70%)	Unipolar depression	MDD+SA vs. MDD+SI-SA	Columbia Classification Algorithm of Suicide Assessment (C-CASA); Kiddie Schedule for Affective Disorders and Schizophrenia; Structured Clinical Interview for DSM-IV Diagnosis (SCID)	sMRI	The following brain regions were found to have greater thickness in subjects with suicidal ideation than in those who had attempted suicide, and thickness was found to be negatively correlated with suicide attempt: right lateral orbitofrontal thickness (*t* = −5.998, *p* < 0.001; *r* = −0.6, *p* < 0.001), left fusiform thickness (*t* = −5.323, *p* < 0.001; *r* = −0.554, *p* < 0.001), left temporal pole volume (*t* = −5.580, *p* < 0.001; *r* = −0.540, *p* < 0.001), left lateral orbitofrontal thickness (*t* = −6.040, *p* < 0.001; *r* = −0.603, *p* < 0.001), left posterior cingulate thickness (*t* = −3.397, *p* < 0.01; *r* = −0.391, *p* = 0.001), right pars orbitalis thickness (*t* = −4.672, *p* < 0.001; *r* = −0.504, *p* < 0.001), right posterior cingulate thickness (*t* = −3.416, *p* < 0.01; *r* = −0.393, *p* = 0.001), and left medial orbitofrontal thickness (*t* = −3.508, *p* < 0.01; *r* = −0.421, *p* < 0.001).
	McLellan [28]	45	12–21 SA: 17.48 ± 1.741 Non-SA: 18.17 ± 1.837	Male (47%) and female (53%)	Unipolar depression (treatment-resistant—failure to respond to ≥8-week SSRI trial)	MDD+SA vs. MDD-SA vs. healthy controls	Kiddie Schedule for Affective Disorders and Schizophrenia (KSADS)	sMRI	Compared with nonsuicidal depressed subjects and healthy controls, the depressed suicide-attempting group exhibited a significant reduction in right superior temporal gyrus (rSTG) volume (*F* = 3.274).
	Pan [26]	44	Suicide attempters 16.2 ± 0.8; MDD w/o suicide attempt 15.9 ± 1.55; healthy controls 15.2 ± 1.4	Male (43%) and female (57%)	Unipolar depression, healthy controls	MDD+SA vs. MDD-SA vs. healthy controls	Columbia Classification Algorithm of Suicide Assessment	Task-based fMRI (facial emotion processing task)	In response to 50% intensity angry faces, suicide attempters showed significantly greater activity than nonattempters in the right anterior cingulate gyrus (*t* = 3.08, *F* = 9.44) and left dorsolateral prefrontal cortex (*t* = 4.33, *F* = 18.51) attentional control circuitry, right primary sensory cortex (*t* = 3.41, *F* = 11.56), and right middle temporal gyrus (*t* = 3.89, *F* = 14.96) and significantly greater activity than healthy controls in the left primary sensory cortex (*t* = 3.64, *F* = 13.16), while nonattempters had significantly lower activity than healthy controls in the right anterior cingulate gyrus (*t* = 3.50, *F* = 12.20) and right ventromedial prefrontal cortex (*t* = 4.72, *F* = 22.01). To neutral faces during the angry emotion processing task, suicide attempters had significantly lower activity than nonattempters in the left fusiform gyrus (*t* = 3.23, *F* = 10.38). Suicide attempters also showed significantly lower activity than healthy controls to 100% intensity happy faces in the left primary sensory cortex (*t* = 3.76, *F* = 14.02) and to neutral faces in the happy run in the right anterior cingulate (*t* = 4.05, *F* = 16.25) and left medial frontal gyrus (*t* = 3.40, *F* = 11.47). Psychophysiological interaction analyses revealed significantly reduced anterior cingulate gyral-insula functional connectivity to 50% intensity angry faces in suicide attempters compared to nonattempters (right insula: *t* = 4.52, *F* = 20.19; left insula: *t* = 3.48, *F* = 12.01) or healthy controls (right insula: *t* = 2.88, *F* = 8.25; left insula: *t* = 4.56, *F* = 20.52).
	Pan [81]	44	Suicide attempters 16.2 ± 0.8, MDD w/o suicide attempt 15.9 ± 1.55, healthy controls 15.2 ± 1.4	Male (43%) and female (57%)	Unipolar depression, healthy controls	MDD+SA vs. MDD-SA vs. healthy controls	Columbia Classification Algorithm of Suicide Assessment	Task-based fMRI (Go/NoGo response inhibition and motor control task)	In a 3-group by 2-condition (“GoNoGo” response inhibition block versus “Go” motor control block) block-design whole-brain analysis, nonsuicide attempters with unipolar depression showed greater activity than depressed suicide attempters in the right anterior cingulate gyrus (*F* = 7.99). Depressed nonattempters, but not depressed attempters, showed significantly greater activity than healthy controls in the left insula (*F* = 9.72) in response to GoNoGo response inhibition blocks.
	Pan [82]	100	13-18	Male and female (percentages not reported)	Unipolar depression, healthy controls	MDD+SA vs. MDD-SA vs. healthy controls	Suicide Intent Scale and Suicide History Form	sMRI	Subjects with unipolar depression and a history of suicide attempt showed significantly reduced cortical volume in the right superior temporal gyrus (rSTG) than healthy controls (*F*-value 6.08, *p* = 0.016).
	Pan [83]	42	MDD suicide attempters 16.2 ± 0.8, MDD w/o suicide attempt 15.9 ± 1.55, healthy controls 15.2 ± 1.4	Male (45%) and female (55%)	Unipolar depression, healthy controls	MDD+SA vs. MDD-SA vs. healthy controls	Columbia Classification Algorithm of Suicide Assessment	Task-based fMRI (Iowa gambling task)	During low-risk decisions in the Iowa gambling task (IGT), depressed nonsuicide attempters, but not depressed attempters, showed significantly greater hippocampal activation than healthy controls (*F* = 16.25). Attempters showed significantly greater activation than healthy controls to low-risk decisions in the left caudate (*F* = 11.98). During high-risk decisions in the IGT, nonattempters showed significantly greater activation than attempters in the right thalamus (*F* = 12.93).
	Zhang [22]	100	15-25 HC: 20.48 ± 1.86 SI: 20.63 ± 3.65 Non-SI: 21.26 ± 3.02	Male (34%) and female 66%	Unipolar depression, healthy controls	MDD+SA vs. MDD-SA vs. healthy controls	History of prior attempt by clinical interview	Resting-state fMRI	Compared with healthy controls, depressed subjects with a history of suicide attempt showed increased connectivity in the left cerebellum (*t* = 2.42), right middle temporal gyrus (*t* = 4.7), right middle occipital gyrus (*t* = 3.31), and left middle frontal gyrus (*t* = 3.56), as well as decreased connectivity in the right posterior cingulate cortex (PCC) (*t* = −3.82). Compared to the nonsuicidal depressed subjects, the suicidal patients showed increased connectivity in the left cerebellum (*t* = 3.33) and the left lingual gyrus (*t* = 3.66), as well as decreased connectivity in the right precuneus (*t* = −4.48).
	Zhang [84]	104	14-25 Mean not reported	Male (34%) and female (66%)	Unipolar depression, healthy controls	MDD+SA vs. MDD-SA vs. healthy controls	Kiddie Schedule for Affective Disorders and Schizophrenia; Structured Clinical Interview for DSM-IV Diagnosis (SCID); Hamilton Depression Rating Scale; Beck Scale for Suicidal Ideation	sMRI	Compared to both healthy controls and subjects with MDD without history of suicide attempts, subjects with MDD with history of suicide attempts had larger bilateral hippocampal fissure volume.
Heterogeneous	Ehrlich [85]	153	14.6 ± 3.4	Male (74%) and female (26%)	Mixed: unipolar depression (52%) and other psychiatric diagnosis not specified (48%)	Heterogeneous +SA vs. heterogeneous-SA	History of prior attempt by clinical interview	sMRI	Deep white matter hyperintensities (DWMH) in the right posterior parietal lobe were observed significantly more frequently in the suicidal group compared to the nonsuicidal group, across all diagnostic groups (*χ*^2^ test, *p* = 0.007). Logistic regression analysis showed an odds ratio of 8.6 (95% CI = 1.3 − 59.0) for positive history of suicide attempt with the presence of parietal lobe DWMHs.
	Ehrlich [86]	153	14.6 ± 3.4	Male (74%) and female (26%)	Mixed: unipolar depression (31.4%), bipolar disorder (22.9%), psychotic disorder NOS (15%), affective disorder NOS (8.5%), conduct disorder/ADHD (11.8%), other psychiatric disorder NOS (9.8%)	Heterogeneous+SA vs. heterogeneous-SA	Modified Pfeffer Rating Scale	sMRI	Subjects with unipolar depression and white matter hyperintensities (WMH) were found to have a significantly higher prevalence of prior suicide attempts compared to subjects without WMH across all diagnostic groups (72% versus 37%; Fisher's exact test, *p* = 0.036). Further logistic regression analysis showed that subjects with unipolar depression who also had WMH were 18.6 times (95% CI = 2.4–145.8) more likely to have had a past suicide attempt when compared with subjects with other psychiatric disorders, or with unipolar depression but no evidence of DWMH.
	Fan [29]	83	14–25 BPD, SA+: 20.3 ± 3 BPD, SA-: 20.2 ± 3.4 MDD, SA+: 18.7 ± 3 MDD, SA-: 18.9 ± 2.7	Male (15%) and female (85%)	Bipolar disorder (55%) and unipolar depression (45%)	Bipolar disorder+SA vs. bipolar disorder–SA vs. MDD+SA vs. MDD-SA	Columbia Suicide History Form	sMRI and DTI	Across and within the bipolar disorder and unipolar depression groups, subjects with a history of suicide attempt showed significantly reduced gray matter volume in the left ventral prefrontal cortex (Brodmann's areas 11 and 47) (*p* < 0.005), as well as reduced fractional anisotropy in the left frontotemporal white matter (including the uncinate fasciculus) (*p* < 0.005), compared to subjects without a history of suicide attempt. Numerical effect sizes were not reported.
	Goodman [87]	26	16.2 ± 0.8 (healthy controls); 15.8 ± 1.1 (comorbid borderline personality disorder and MDD)	Male (23%) and female (77%)	Subjects meeting criteria for both borderline personality disorder and MDD (50%); healthy controls (50%)	MDD+bipolar disorder vs. healthy controls	Lifetime Self-Destructiveness Scale (LSDS)	sMRI	Among the adolescent subjects with comorbid borderline personality disorder and MDD, the number of prior suicide attempts was associated with smaller overall volume of BA24 of the anterior cingulate gyrus (*r* = −0.40, *p* = 0.044) (averaged across gray and white matter), but not with the volume for any of the other anterior cingulate BAs assessed (BA25, BA23, BA29, and BA31). Among this group, a greater number of suicide attempts were also associated with greater white matter volume (*r* = 0.39, *p* = 0.049) but not gray matter volume in BA23 of the anterior cingulate.
	Huber [25]	63	13–21 Controls: 18.56 ± 2.42 BD, SA-: 17.44 ± 2.48 BD, SA+: 17.47 ± 2.45	Male (38%) and female (62%)	Bipolar disorder, healthy controls	Bipolar disorder vs. Healthy controls	Columbia Suicide Severity Rating Scale	sMRI	Bipolar youth with a history of suicide attempt showed reduced left lateral orbitofrontal cortex (OFC) volumes compared to healthy controls (*F*-value 5.94, *p* = 0.005), but there was no significant difference between bipolar suicide attempters and bipolar nonattempters. Controls and bipolar nonattempters had significantly greater left (*F*-value 4.44, p =0.017) and right (*F*-value 13.11, *p* < 0.001) lateral OFC cortical thickness than bipolar attempters.
	Johnston [19]	68	14–25 BD, SA+: 20.5 ± 3 BD, SA-: 20.6 ± 3.2	Male (37%) and female (63%)	Bipolar disorder	Bipolar disorder+SA vs. Bipolar-SA	Columbia Suicide History Form	sMRI, DTI, and task-based fMRI (facial emotion processing task)	Compared to the group without history of suicide attempt, the suicide-attempting group showed reductions in gray matter volume in the right orbitofrontal cortex (*t* = 4.16), right hippocampus (*t* = 4.23), and bilateral cerebellum (*t* = 3.35); white matter fractional anisotropy in the uncinate fasciculus (*t* = 4.78), left uncinate/ventral prefrontal region (*t* = 3.79), and right cerebellum (*t* = 4.06); and amygdala functional connectivity to left ventral prefrontal (happy face processing task (*t* = 4.31), neutral face processing task (*t* = 4.21), and fearful face processing task (*t* = 4.11)) and right rostral prefrontal cortex (neutral face processing task (*t* = 4.22)).

Abbreviations: ADHD = attention-deficit/hyperactivity disorder; BPD = bipolar disorder; DTI = diffusion tensor imaging; fMRI = functional magnetic resonance imaging; GAD = generalized anxiety disorder; HC = healthy controls; MDD = major depressive disorder; PTSD = posttraumatic stress disorder; RSFC = resting-state functional connectivity; SA = suicide attempt; SI = suicidal ideation; sMRI = structural magnetic resonance imaging.

**Table 2:** Brain regions reported in the reviewed studies, organized by diagnosis (major depressive disorder (MDD) vs. other conditions or heterogeneous groups) and by suicidal ideation (SI) vs. attempt (SA). This resulted in the following four categories: MDD+SI, MDD+SA, heterogeneous+SI, and heterogeneous+SA. In this descriptive approach, we indicate next to each brain region the frequency with which this region was reported as a finding.

Diagnosis	Suicidal ideation	Suicidal attempt
MDD	(i) Amygdala (2) (ii) Anterior cingulate (1) (iii) Cerebellum (3) (iv) Cuneus (1) (v) Dorsolateral prefrontal cortex (2) (vi) Fusiform gyrus (1) (vii) Hippocampus (3) (viii) Inferior frontal gyri (2) (ix) Insula (2) (x) Medial prefrontal cortex (3) (xi) Middle frontal gyri (2) (xii) Midline cortical structure (1) (xiii) Occipital gyrus (2) (xiv) Postcentral gyri (2) (xv) Posterior cingulate cortex (2) (xvi) Posterior parietal cortex (1) (xvii) Precentral gyrus (3) (xviii) Precuneus (3) (xix) Superior frontal gyri (2) (xx) Superior parietal lobe (1) (xxi) Thalamus (1)	(i) **Amygdala (2)** (ii) Anterior cingulate (3) (iii) **Cerebellum (3)** (iv) Dorsolateral prefrontal cortex (2) (v) Fusiform (2) (vi) **Hippocampus (1)** (vii) Insula (1) (viii) **Lateral orbitofrontal cortex (1)** (ix) Medial prefrontal cortex (2) (x) Medial orbitofrontal (1) (xi) **Middle temporal gyrus (2)** (xii) Occipital gyrus (2) (xiii) Periventricular white matter (1) (xiv) Postcentral gyri (1) (xv) Posterior cingulate (2) (xvi) Precuneus (2) (xvii) Superior frontal gyrus (1) (xviii) **Superior temporal gyrus, incl. temporal pole (3)** (xix) **Uncinate fasciculus (frontotemporal white matter) (1)**

Heterogeneous	(i) Caudate (1) (ii) Cerebellum (1) (iii) Dorsolateral prefrontal cortex (2) (iv) Frontoparietal (1) (v) Insula (1) (vi) Occipital lobe (1) (vii) Orbitofrontal cortex (1) (viii) Pallidum (1) (ix) Precentral gyrus (1) (x) Putamen (1) (xi) Superior temporal sulcus left bank (1) (xii) Temporal pole (1) (xiii) Temporoparietal junction (1) (xiv) Thalamus (1) (xv) Ventral diencephalon (1)	(i) **Amygdala (1)** (ii) **Cerebellum (1)** (iii) **Hippocampus (1)** (iv) **Lateral orbitofrontal cortex (2)** (v) Posterior parietal lobe deep white matter (1) (vi) Rostral prefrontal cortex (1) (vii) **Uncinate fasciculus (frontotemporal white matter) (2)** (viii) Ventral prefrontal cortex (3)

Bold: convergent findings in attempters across studies and diagnostic groups, since suicide is a transdiagnostic behavior, and attempters are the most crucial category of patients for suicide prevention (categories MDD+SA and heterogeneous+SA).

## Data Availability

The neuroimaging data supporting this systematic review are from previously reported studies and datasets, which have been cited. The processed data are available from the corresponding author upon request.
